# Prevalence and modifiable risk factors for pediatric flatfoot among schoolchildren in Kunming and Kandahar: a cross-sectional study

**DOI:** 10.3389/fped.2025.1739543

**Published:** 2026-01-20

**Authors:** Abdul Waheed Bahir, Munir Ahmad Bahir, Karthikesu Kartheepan, Qudratullah Bahir, Gan Xuewen, Gu Shao, Xiao Jiayu, Xiong Ying

**Affiliations:** 1 Department of Orthopedic Surgery, Yan’an Hospital Affiliated to Kunming Medical University, Kunming, Yunnan, China; 2 Department of Clinical Medicine, Malalay University, Kandahar, Afghanistan; 3 School of Public Health, Kunming Medical University, Kunming, Yunnan, China

**Keywords:** cross-sectional study, pediatric flatfoot, prevalence, risk factors, schoolchildren

## Abstract

**Background:**

Pediatric flatfoot is a prevalent musculoskeletal condition that may impair gait patterns, posture, and quality of life. Despite its clinical importance, only a few studies have explored how its prevalence and risk factors vary across different sociocultural settings. To date, no study has directly compared pediatric flatfoot between the two countries. Therefore, this study aimed to determine the prevalence of pediatric flatfoot and identify the associated modifiable risk factors among schoolchildren from two different countries representing urbanized and resource-limited settings.

**Methods:**

A cross-sectional study was conducted between December 2023 and February 2025 among the schoolchildren aged 7–14 years with a total number of 4,205 in Kunming, China, and Kandahar, Afghanistan. Foot morphology was assessed using an optical podoscope, and flatfoot was classified using a line-based footprint method. Anthropometric data and information on footwear, physical activity, and foot pain were collected using standardized questionnaires. Logistic regression analysis was used to identify the independent risk factors.

**Results:**

The overall prevalence of flatfoot was 12.8%, with 11.0% in Kunming and 14.6% in Kandahar. Flatfoot is more common in boys, children aged 11–14 years, and urban residents. Obesity, insufficient physical activity, closed-toe footwear, and foot pain were significantly associated with higher odds of the flatfoot with consistent patterns across both sites. Most cases were bilateral, and approximately one-fifth were classified as severe.

**Conclusion:**

Pediatric flatfoot remains a widespread condition among school-aged children in both urban and resource-limited settings. Its strong and consistent links with modifiable factors such as body mass index, physical activity, footwear, and foot pain highlight the need for early school-based screening and prevention programs that encourage active lifestyles, healthy body weight, and the use of proper footwear. These results offer valuable cross-cultural insights to support pediatric foot health and guide future preventive initiatives.

## Introduction

The human foot provides the structural foundation for upright posture and locomotion. It transmits forces between the body and the ground through 26 bones, 33 joints, and more than 100 muscles, ligaments, and tendons (1). The longitudinal and transverse arches of the foot play an important role in balance creation and efficient movement. The medial longitudinal arch (MLA) is particularly important for distributing the weight and absorbing the shock (2). Pediatric flatfoot or pes planus is defined by the collapse or absence of the MLA, which produces partial or complete sole-to-ground contact. The two clinical patterns are recognized: flexible flatfoot, in which the arch reappears when non-weight bearing, and rigid flatfoot, in which the arch remains absent regardless of position and is more often associated with the underlying pathology (3). Flatfoot is common in children whose skeletal structures are still developing. However, the MLA continues to mature through the first decade of life (4). Most cases of flexible flatfoot in children are physiologic and typically resolve spontaneously with growth. These cases are generally painless and do not impair function. However, a subset of children exhibit persistent, symptomatic, or rigid flatfoot, which can be associated with discomfort, altered gait, postural imbalance, and long-term complications such as ankle or knee pain, plantar fasciitis, and reduced quality of life (5, 6). Without intervention, persistent cases rarely resolve spontaneously and may progressively worsen, highlighting the importance of early recognition and management (7). The Prevalence estimates vary by the age and assessment method; ∼21%–57% in children aged 2–6 years, ∼13.4%–27.6% in primary-school children, and ∼5%–14% in adults (8). Consistent risk factors include male sex, younger age, and elevated body mass index (BMI) (9, 10). Urban environments are often associated with lower physical activity levels and more structured footwear use, factors that have been identified as contributors to weaker foot musculature and an increased risk of pediatric flatfoot (11). Within this context, Kunming represents an urbanized setting included in the present study. Conversely, in resource-limited areas such as Kandahar, limited access to appropriate footwear and preventive care presents distinct developmental challenges. This contrast highlights the importance of shoe practices; empirical evidence indicates that children who spend a greater proportion of time barefoot have a lower prevalence of flatfoot compared with those who begin wearing shoes at an earlier age (12). Despite a growing body of literature, no comparative study has used standardized school-based screening across the two countries to examine the associated factors and similarities. The current study aims to address this gap by evaluating schoolchildren who are between 7 and 14 years old in Kunming, China, and Kandahar, Afghanistan. These two groups differ in several aspects such as urbanization, socioeconomic status, footwear habits, and access to medical facilities. Using an optical podoscope with a line-based footprint classification, we estimate the prevalence of flatfoot and assess associations with demographic, anthropometric, and lifestyle factors (age, gender, residence, BMI, footwear, physical activity, and foot pain). We aim to provide comparable evidence across the urbanized and resource-limited environments.

## Methods

### Study area

The study was conducted in Kunming, Yunnan Province, China, and Kandahar, Kandahar Province, Afghanistan. These locations were selected to allow direct comparison between an urbanized setting (Kunming) and a resource-limited context (Kandahar).

### Study design and population

This school-based, cross-sectional comparative study was conducted from 13 December 2023 to 25 February 2025, among primary-school children aged 7–14 years. All enrolled students within this age range in the selected schools were eligible. The exclusion criteria were congenital foot deformities other than flatfoot, prior lower-limb surgery, or lack of consent/assent. This study was conducted in accordance with the Declaration of Helsinki and was reviewed and approved by the Ethics Committee of Yan'an Hospital affiliated to Kunming Medical University, China (Approval No. 2025-339-01), and by the Ethics Committee of Mohmand Hospital, Malalay University, Kandahar, Afghanistan (Approval No. 345-M-KD334). The objectives of the study were outlined, and privacy and confidentiality were guaranteed before participation. Consent with parents/guardians was written, and consent with children was obtained. All procedures were conducted in line with the applicable rules and standards.

### Sampling procedure and sample size

Schools were selected using stratified random sampling to ensure the proportional representation of urban and rural settings. In Kunming, 2,200 eligible students from 7 schools (4 urban, 3 rural) were included; in Kandahar, 10 schools (6 urban, 4 rural) were included (2,200 eligible students). A total of 4,400 children were eligible; 2,050 from Kunming and 2,155 from Kandahar completed the study, with a total of 4,205 participants, and the participation rate was 95.6%.

### Pilot study

Before the main data collection, a pilot study of 50 participants was conducted to assess the clarity of the questionnaire, test the feasibility of the measurement procedures, and evaluate inter-observer reliability among examiners. The children included in the pilot were excluded from the final analysis. Inter-observer agreement was high with Cohen's kappa values exceeding 0.80 indicating excellent reliability.

### Data collection tools

**Figure 1:**
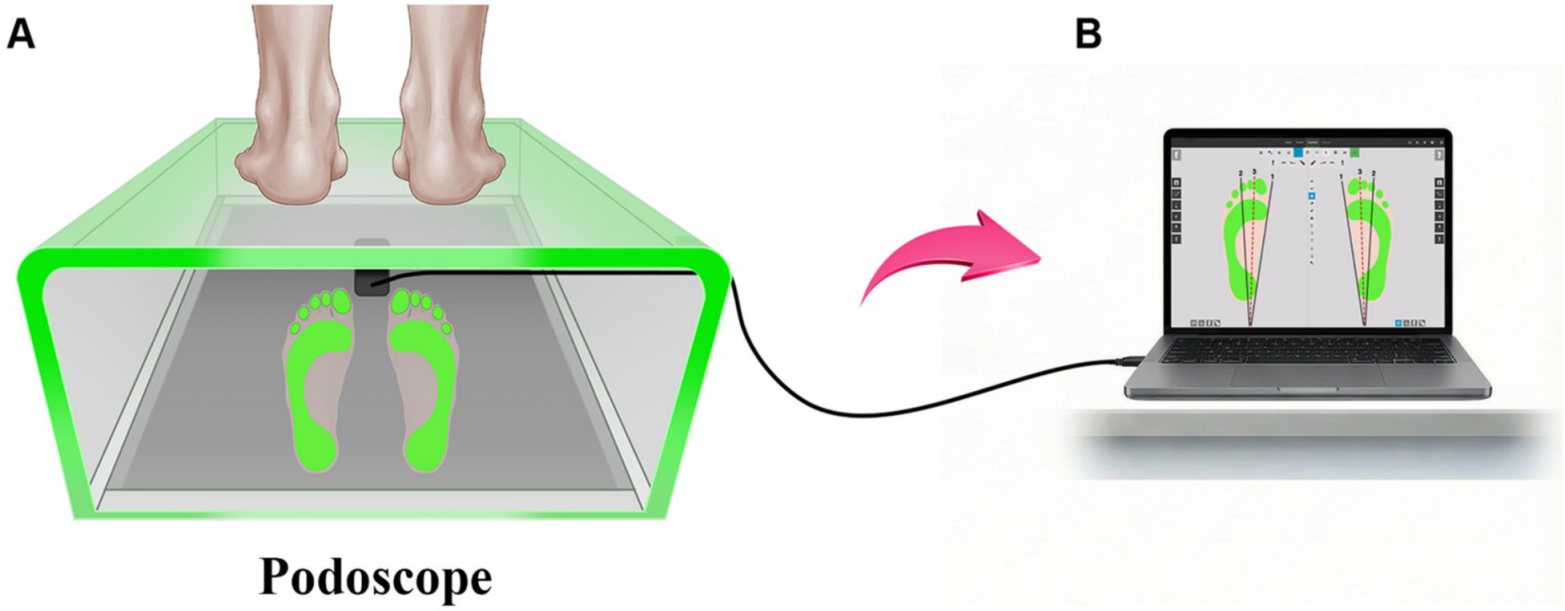
Podoscope-based plantar image acquisition process. **(A)** A participant stands barefoot on an LED podoscope with the plantar surface clearly visible through the mirrored base. **(B)** The reflected plantar images are transferred to a computer for assessment of foot arch characteristics.

A structured, pilot-tested questionnaire was used to collect sociodemographic characteristics, physical activity, footwear habits, and foot pain. Anthropometry used a calibrated stadiometer (0.1 cm) and digital scale (0.1 kg). Measurements were taken with children barefoot and in light clothing. Plantar images were obtained using a podoscope, an optical foot assessment, and a recording device equipped with an LED perimeter and a mirrored base (Figure 1).

### Data collection procedures

**Figure 2:**
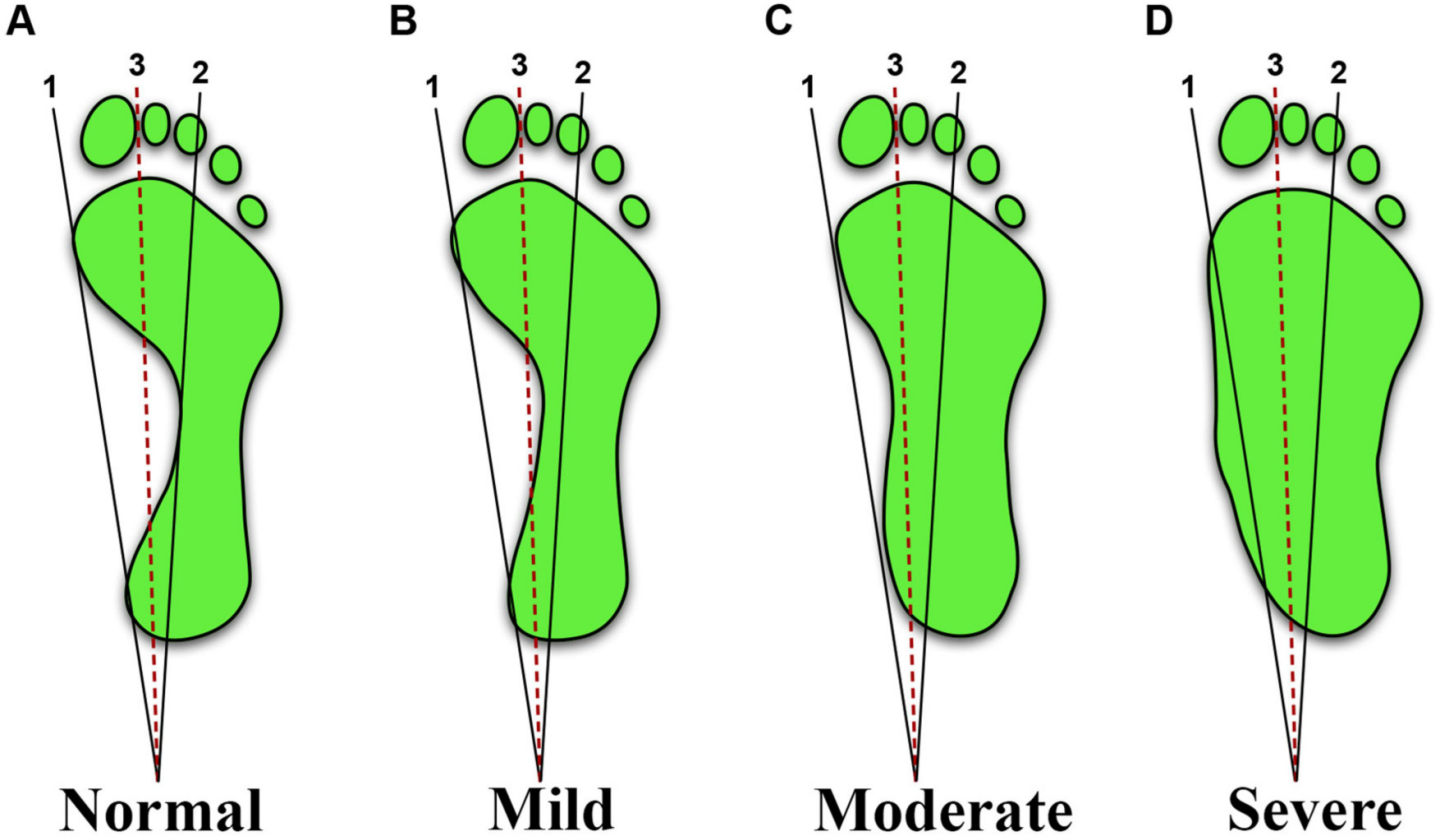
Schematic illustration of the line-based footprint method for assessing flatfoot severity. **(A)** Normal, **(B)** mild, **(C)** moderate, and **(D)** severe.

Children stood barefoot on the podoscope in a standardized bipedal stance. Weight was distributed evenly, and the children maintained a forward gaze. The device captured a clear plantar image for each foot. On each image, three reference lines were drawn: (1) a medial tangent from the great toe to the heel, (2) a line from the third toe to the heel center, and (3) the angle bisector of lines 1 and 2 (13). No discomfort or adverse events were recorded and addressed per protocol. Flatfoot was classified according to the medial arch position relative to three reference lines (Figure 2) as follows. Normal arch: medial arch edge does not cross line (2). Mild flatfoot: medial arch edge lies between lines 2 and 3. Moderate flatfoot: medial arch edge lies between lines 1 and 3. Severe flatfoot: medial arch edge crosses line 1 (13). Severity was assigned per foot, and laterality (unilateral/bilateral) was recorded. Children were considered flatfooted if either foot met the criteria, as unilateral flatfoot is clinically relevant and is commonly included in prevalence estimates in school-based screening studies.

### Operational definitions

Physical activity was categorized as sufficient (≥180 min per week) or insufficient (<180 min per week) (14). Footwear type was defined as closed-toe shoes (covering the entire foot, including the heel) or sandals (a sole secured by straps). BMI categories followed the WHO 2007 BMI-for-age standards: underweight (greater than −2 SD), normal (−2 SD to +1.99 SD), overweight (+1 SD to +2 SD), and obese (less than +2 SD) (15). Flatfoot status used the line-based method described above. A child was considered flatfooted if either foot met the flatfoot criteria, with severity graded as mild, moderate, or severe, and laterality (unilateral/bilateral) recorded.

#### Study variables

The dependent variable was flatfoot, whereas the independent variables included age, sex, residence (urban/rural), BMI, footwear type, physical activity, and foot pain.

### Quality control

All data were collected by the trained medical staff under the supervision of orthopedic specialists. Standardized training sessions and the pilot study ensured the feasibility of the tool and inter-rater agreement. Standardized manuals, identical data collection forms, and regular monitoring visits helped maintain uniformity across sites. The anthropometric equipment was calibrated weekly during the study period.

### Data processing

At the end of each day, questionnaires and measurement sheets were checked for completeness and internal consistency. Data were then coded and entered twice into SPSS v29.0 (IBM, Armonk, NY, USA). Supervisors reviewed and resolved any discrepancies. Once resolved, a de-identified analysis dataset was locked before modeling.

### Statistical analysis

Categorical variables were summarized as counts and percentages, whereas continuous variables were summarized as means (SD) or medians (IQR) after assessing normality (Shapiro–Wilk). Group differences were tested with chi-squared tests. Associations with flatfoot were examined using binary logistic regression, first bivariable and then multivariable, reporting crude and adjusted odds ratios (95% CIs). The multivariable model included age, sex, BMI, residence, and site *a priori*, with additional covariates retained if bivariable *p* ≤ 0.25. Model fit and assumptions were assessed using the Hosmer–Lemeshow test and variance inflation factors. Standard errors were cluster-robust at the school level. Primary analyses used complete-case data, and statistical significance was set at two-tailed *p* < 0.05. Analyses were performed in SPSS v29.0.

## Results

A total of 4,205 schoolchildren were examined, including 2,050 from Kunming, China, and 2,155 from Kandahar, Afghanistan. The overall participation rate was 95.6%. The baseline demographic and lifestyle characteristics are summarized in Table 1. The distribution of sex differed between the sites with a higher proportion of boys in Kandahar than in Kunming (*p* < 0.05). The older children group (11–14 years) was more represented in Kandahar (51.9%) than in Kunming (47.4%; *p* < 0.05). BMI categories also varied significantly (*p* < 0.05): underweight was more common in Kandahar, whereas overweight and obesity were slightly more frequent in Kunming. Urban residence predominated in Kunming at 75.2% compared with Kandahar at 62.7%. Moreover, footwear patterns showed striking differences between the two groups (*p* < 0.05). Most children in Kunming wore closed-toe shoes (70.6%), whereas sandals were predominant in Kandahar with 61.9%. No significant differences were observed between the two sites in physical activity (≥180 min/week) or reported foot pain in the preceding 6 months (both *p* > 0.05).

**Table 1:** Baseline characteristics of schoolchildren in Kunming and Kandahar.

Variable	Kunming (*n* = 2,050) (%)	Kandahar (*n* = 2,155) (%)	Total (*n* = 4,205) (%)	*p*-value
Gender—*n* (%)				0.013
Boys	958 (46.7)	1,091 (50.6)	2,049 (48.7)	
Girls	1,092 (53.3)	1,064 (49.4)	2,156 (51.3)	
Age—years				
	Median 10	Median 11	Median 10	
	IQR: 8–12	IQR: 8–13	IQR: 8–12	
Age group—*n* (%)				0.003
7–10 years	1,079 (52.6)	1,036 (48.1)	2,115 (50.3)	
11–14 years	971 (47.4)	1,119 (51.9)	2,090 (49.7)	
BMI (kg/m^2^)				
	Median 16.8	Median 16.1	Median 16.4	
	IQR: 15.5–18.2	IQR: 14.8–17.5	IQR: 15.1–17.9	
BMI categories (WHO 2007)—*n* (%)				<0.0001
Underweight	189 (9.2)	404 (18.7)	593 (14.1)	
Normal	1,372 (66.9)	1,318 (61.2)	2,690 (64.0)	
Overweight	323 (15.8)	267 (12.4)	590 (14.0)	
Obese	166 (8.1)	166 (7.7)	332 (7.9)	
Residence—*n* (%)				<0.0001
Urban	1,541 (75.2)	1,351 (62.7)	2,892 (68.8)	
Rural	509 (24.8)	804 (37.3)	1,313 (31.2)	
Footwear type—*n* (%)				<0.0001
Sandals	603 (29.4)	1,334 (61.9)	1,937 (46.1)	
Closed-toe	1,447 (70.6)	821 (38.1)	2,268 (53.9)	
Physical activity—*n* (%)				0.300
Sufficient activity (≥180 min/week)	1,639 (80.0)	1,694 (78.6)	3,333 (79.2)	
Insufficient activity (<180 min/week)	411 (20.0)	461 (21.4)	872 (20.8)	
Foot pain (past 6 months)—*n* (%)				0.317
Yes	339 (16.5)	331 (15.4)	670 (15.9)	
No	1,711 (83.5)	1,824 (84.6)	3,535 (84.1)	

IQR, interquartile range; BMI, body mass index; kg/m^2^, kilograms per square meter.

The overall prevalence of the flatfoot was 12.8%, 540/4,205 with a higher prevalence in Kandahar compared with Kunming (14.6% vs. 11.0%). Flatfoot prevalence was greater among boys than girls in Kandahar with 18.2%, while the sex difference was less pronounced in Kunming with 12.3% (overall *p* < 0.05). Prevalence was also higher in the older children group (11–14 years), compared with the younger children group in both sites (*p* < 0.05). Urban children were more frequently affected than rural children in both Kunming (11.9% vs. 8.3%) and Kandahar (17.0% vs. 10.7%; *p* < 0.05).

BMI showed a clear dose–response relationship: the obese children had the highest prevalence at 26.5% in Kunming and 27.1% in Kandahar, overweight children had an intermediate prevalence, whereas underweight children had the lowest (*p* < 0.05). Children with foot pain in the past 6 months were substantially more likely to have the flatfoot (Kunming, 27.7% vs. 7.7%; Kandahar, 33.8% vs. 11.1%; *p* < 0.05). Insufficient physical activity was also associated with the higher prevalence (Kunming, 19.7% vs. 8.8%; Kandahar, 25.2% vs. 11.7%; *p* < 0.05). Closed-toe footwear was significantly associated with a higher prevalence compared with sandals in both sites (Kunming, 12.6% vs. 7.1%; Kandahar, 16.9% vs. 13.2%; *p* < 0.05; Table 2).

**Table 2:** Distribution of flatfoot and normal foot by demographic, anthropometric, lifestyle, and clinical characteristics.

Variable	Category	Kunming normal foot (*n* = 1,825) (%)	Kunming flat foot (*n* = 225) (%)	Kandahar normal foot (*n* = 1,840) (%)	Kandahar flat foot (*n* = 315) (%)	*p*-value
Gender—*n* (%)	Boys	840 (87.7)	118 (12.3)	892 (81.8)	199 (18.2)	<0.0001
	Girls	985 (90.2)	107 (9.8)	948 (89.1)	116 (10.9)	
Age group—*n* (%)	7–10 years	982 (91.0)	97 (9.0)	886 (85.5)	150 (14.5)	0.026
	11–14 years	843 (86.8)	128 (13.2)	954 (85.3)	165 (14.7)	
Residence—*n* (%)	Urban	1,358 (88.1)	183 (11.9)	1,122 (83.0)	229 (17.0)	<0.0001
	Rural	467 (91.7)	42 (8.3)	718 (89.3)	86 (10.7)	
BMI—*n* (%)	Underweight	178 (94.2)	11 (5.8)	358 (88.6)	46 (11.4)	<0.0001
	Normal	1,258 (91.7)	114 (8.3)	1,155 (87.6)	163 (12.4)	
	Overweight	267 (82.7)	56 (17.3)	206 (77.2)	61 (22.8)	
	Obese	122 (73.5)	44 (26.5)	121 (72.9)	45 (27.1)	
Foot pain—*n* (%)	Yes	245 (72.3)	94 (27.7)	219 (66.2)	112 (33.8)	<0.0001
	No	1,580 (92.3)	131 (7.7)	1,621 (88.9)	203 (11.1)	
Physical activity—*n* (%)	Sufficient	1,495 (91.2)	144 (8.8)	1,495 (88.3)	199 (11.7)	<0.0001
	Insufficient	330 (80.3)	81 (19.7)	345 (74.8)	116 (25.2)	
Footwear type—*n* (%)	Closed-toe	1,265 (87.4)	182 (12.6)	682 (83.1)	139 (16.9)	0.007
	Sandals	560 (92.9)	43 (7.1)	1,158 (86.8)	176 (13.2)	

Among children, the flatfoot severity distributions were comparable between both sites. In Kunming, 35.6% were mild, 44.4% moderate, and 20.0% severe; in Kandahar, 34.9% were mild, 46.0% moderate, and 19.0% severe. Bilateral involvement was predominant at 85.3% in Kunming and 88.3% in Kandahar, with unilateral involvement less frequent (14.7% and 11.7%, respectively). No significant inter-site differences were found in severity or in laterality.

Multivariable logistic regression analyses (Table 3) identified several independent risk factors. Older age (11–14 years) was associated with increased odds of flatfoot in Kunming [adjusted odds ratio (AOR) 1.33, 95% CI: 1.01–1.75, *p* < 0.05], but not in Kandahar (AOR 1.11, 95% CI: 0.88–1.39, *p* > 0.05). Male sex was also a risk factor in Kunming (AOR 1.36, 95% CI: 1.03–1.79, *p* < 0.05), whereas no sex difference was observed in Kandahar (AOR 1.20, 95% CI: 0.94–1.54, *p* > 0.05). Urban residence was associated with higher odds in both sites (Kunming: AOR 1.45, 95% CI: 1.00–2.10; Kandahar: AOR 1.60, 95% CI: 1.20–2.13; pooled *p* < 0.05). BMI demonstrated a strong dose–response effect: overweight and obese children had 1.7–3.2 times higher odds compared with normal-weight peers (*p* < 0.05). Foot pain was one of the strongest predictors, associated with approximately threefold higher odds (Kunming AOR 3.00, Kandahar AOR 2.80; *p* < 0.05). Insufficient physical activity was also strongly associated (AOR ∼2.0 in both sites; *p* < 0.05). Finally, closed-toe footwear was consistently associated with a greater risk compared with sandals (Kunming AOR 1.80; Kandahar AOR 1.84; *p* < 0.05).

**Table 3:** Factors associated with flatfoot by site: crude and adjusted odds ratios.

Variable	Category	Kunming COR (95% CI)	Kunming AOR (95% CI)	Kandahar COR (95% CI)	Kandahar AOR (95% CI)	*p*-value
Age group	7–10 years	1	1	1	1	–
	11–14 years	1.54 (1.16–2.04)	1.33 (1.01–1.75)	1.02 (0.80–1.29)	1.11 (0.88–1.39)	0.041
Gender	Girls	1	1	1	1	–
	Boys	1.29 (0.98–1.70)	1.36 (1.03–1.79)	1.83 (1.43–2.34)	1.20 (0.94–1.54)	0.001
Residence	Rural	1	1	1	1	–
	Urban	1.50 (1.06–2.14)	1.45 (1.00–2.10)	1.71 (1.31–2.23)	1.60 (1.20–2.13)	0.016
BMI	Normal	1	1	1	1	–
	Underweight	0.68 (0.36–1.29)	0.70 (0.40–1.22)	0.80 (0.57–1.13)	0.85 (0.60–1.19)	0.332
	Overweight	2.35 (1.66–3.32)	1.90 (1.35–2.68)	2.10 (1.51–2.92)	1.70 (1.22–2.38)	0.002
	Obese	4.02 (2.71–5.97)	3.20 (2.10–4.90)	2.64 (1.80–3.85)	2.10 (1.45–3.05)	<0.001
Foot pain	No	1	1	1	1	–
	Yes	4.61 (3.42–6.20)	3.00 (2.10–4.28)	4.11 (3.13–5.38)	2.80 (2.00–3.92)	<0.001
Physical activity	Sufficient	1	1	1	1	–
	Insufficient	2.56 (1.90–3.44)	1.95 (1.40–2.72)	2.54 (1.96–3.28)	2.05 (1.55–2.71)	<0.001
Footwear type	Sandals	1	1	1	1	–
	Closed-toe	1.87 (1.32–2.65)	1.80 (1.23–2.62)	1.34 (1.05–1.71)	1.84 (1.33–2.43)	0.006

COR, crude odds ratio; AOR, adjusted odds ratio; CI, confidence interval.

## Discussion

Pediatric flatfoot is common in school-aged children although reported prevalence varies depending on age structure, case definitions, and measurement methods. In this school-based comparison from Kunming (China) and Kandahar (Afghanistan), the overall prevalence was 12.8% with higher rates in Kandahar (14.6%) than in Kunming (11.0%). Kunming, the capital of Yunnan Province, is a high-altitude urban center with relatively structured school systems, greater access to healthcare, and widespread use of closed-toe footwear. In contrast, Kandahar is a southern Afghan city with a hotter and drier climate, where sandals and periods of barefoot walking are common, and preventive health infrastructure is more limited. To our knowledge, this is the first study to directly compare pediatric flatfoot between China and Afghanistan. No prior research has provided such a cross-country analysis. This approach provides unique benefits by distinguishing globally relevant determinants of the flatfoot from context-specific influences shaped by the environment, culture, and resources. Importantly, our experience also demonstrates that podoscope-assisted plantar image acquisition is feasible in Kandahar real-world school settings, providing standardized images that enable accurate line-based flatfoot classification, which in turn can directly inform school-based prevention efforts. This small difference shows that factors such as environment, lifestyle, and culture can still influence how common flatfoot is even when the same diagnostic method is used. Across both sites, urban residence, higher BMI, lower physical activity, closed-toe footwear, and foot pain in the previous 6 months were consistently associated with the flatfoot. These findings highlight the importance of the preventive strategies. In both locations, especially in Kandahar, access to preventive care is limited, and school-based programs that promote healthy body weight, encourage regular physical activity, teach safe footwear practices, and address foot pain early could have a significant public health impact. These modifiable correlates are directly relevant to the school health services in diverse cultural settings.

Age and sex-related patterns were not the same across the two study sites. Although many earlier studies have shown that the flatfoot tends to decrease as children get older, our results suggest that this pattern may depend on local conditions. In Kunming, the older children group (11–14 years) was more likely to have flatfoot, while in Kandahar, there was no clear difference between age groups. This difference suggests that the local factors, such as footwear choices, daily activity levels, growth rate, and even the type of flooring, may influence how age affects the development of flatfoot (16). Similarly, although the boys generally show a higher prevalence of flatfoot than girls, our findings also indicate that this pattern varies by location. After adjustment, sex differences were apparent in Kunming but not in Kandahar. This suggests that the gender related variations in flatfoot are not universal and may be shaped by cultural differences in physical activity, growth patterns, or footwear habits. In both populations, BMI showed a clear trend: overweight and obese children were increasingly more likely to have the flatfoot. This finding aligns with the existing biomechanical and epidemiological evidence that excess body weight increases pressure on the feet and contributes to the flattening of the arch (17). The consistent link between the BMI and the flatfoot across two culturally different populations supports BMI as a universal risk factor for pediatric flatfoot. Footwear also appeared to play an important role; those children who wore closed-toe shoes had a higher likelihood of developing flatfoot compared with those who wore sandals. This finding agrees with the previous research suggesting that the rigid or less flexible footwear may restrict arch development and the foot muscle function, while softer or minimal footwear may encourage the stronger arch formation (18).

Because the footwear was grouped broadly as either closed-toe shoes or sandals, these findings reflect the general differences between categories rather than specific design, fit, or duration of wear and should therefore be interpreted with caution. It is worth noting that most children in Kunming wore closed shoes, while sandals were more common in Kandahar. Despite these cultural contrasts, the association between the footwear type and flatfoot was consistent across both sites. This consistency indicates that the footwear is likely to have a direct effect on how the arch develops rather than just reflecting the differences in lifestyle or climate.

Physical activity showed a protective effect, with those children who were less active having higher odds of flatfoot. This supports the idea that regular walking, play, and engagement of the foot intrinsic muscles also help maintain a healthy arch structure. Children who reported foot pain had a much higher prevalence of flatfoot, which is consistent with earlier studies showing that flatfoot can negatively affect quality of life and lead to other foot problems, even in children who were once thought to be “asymptomatic” (19).

The strong association between foot pain and flatfoot in both sites highlights the need to avoid dismissing pediatric flatfoot as purely physiological or harmless. Most of the identified cases were bilateral, and approximately 20% were classified as severe based on the line-based footprint method. The similarity in the severity and the laterality between the Kunming and Kandahar indicates that once the flatfoot develops, its clinical characteristics are largely consistent across the populations, even when environmental factors differ. Although evaluating treatment was beyond the scope of this study, existing evidence supports conservative approaches such as maintaining a healthy weight, modifying physical activity, strengthening the intrinsic foot muscles, and using orthotic devices judiciously and for limited periods to improve outcomes in the symptomatic children (20).

Placing our findings in an international context, our estimates sit within the global spread for school-age children. In Iran, school-based studies report a prevalence of approximately 17%, with flatter feet more common at younger ages, consistent with developmental consolidation of the MLA (16). In Colombia, a two-city sample (ages 3–10) found ∼15.7%, higher in younger strata and varying by city, underscoring how age structure and urban context shape absolute rates (21). In Saudi Arabia, several regional studies using footprint/posture indices report ∼25%–30% among schoolchildren, highlighting heterogeneity by instrument and sampling frame (22). In Taiwan, cohorts enriched with younger primary-school ages show higher detection, with one large study identifying elevated odds in 7–9-year-olds and strong associations with male sex and overweight/obesity, illustrating how age mix and BMI shift prevalence (23). A meta-analytic view also suggests a pooled detection rate of approximately 25%, with higher odds in boys and a declining prevalence with age (11).

Overall, these comparisons position Kunming (11.0%) toward the lower-middle range of the global estimates and Kandahar (14.6%) closer to the mid-range, which emphasizes that factors such as the study methods, age distribution, footwear habits, and levels of urbanization can strongly influence the prevalence rates. The slightly higher prevalence in Kandahar may reflect the nutritional transitions, a mix of the underweight and overweight patterns, differences in the footwear availability, and the limited access to preventive healthcare. In contrast, the lower prevalence in Kunming may be linked to the structured school-based activity programs and better access to the pediatric screening services.

This study has several strengths, including its large sample size *n* = 4,205, the high participation rate, pre-specified analysis plan, cluster-robust modeling by school, and standardized podoscope imaging conducted in two culturally diverse settings. The consistency of the key associations across both sites highlights the practicality of implementing school-based initiatives that combine the BMI monitoring and simple foot posture screening, promotion of regular physical activity, and context-appropriate footwear recommendations that focus on flexibility, comfort, and safety. Growing evidence that the flexible footwear and the targeted foot-core strengthening enhance muscle strength, balance, and arch development supports the value of such population-level preventive approaches (24).

### Limitations

The cross-sectional design precludes causal inference. Footprint-based classification, although practical for school screening, may misclassify borderline cases in relation to radiographic criteria. Exposures such as footwear and activity were captured in simplified categories and did not account for habitual barefoot time, shoe fit, flooring, hypermobility/ligamentous laxity, or wear time; self-reported activity and pain are subject to recall bias. Data collection spanned from 13 December 2023 to 25 February 2025. Although procedures were standardized, unmeasured seasonal or cohort effects (e.g., school schedules and weather-related activities) cannot be excluded. Nevertheless, the consistency of risk factor directions across both populations strengthens the validity and generalizability of the associations.

## Conclusion

Pediatric flatfoot was common in both Kunming and Kandahar, with prevalence slightly higher in Kandahar. Most cases were bilateral, and approximately one-fifth were severe. Consistent associations with BMI, physical activity, footwear, and foot pain across both sites suggest that these are globally relevant determinants of flatfoot and should be central to prevention efforts. In contrast, age- and sex-related associations varied between sites, showing that demographic influences are context-dependent rather than universal. Importantly, this study represents the first direct cross-country comparison of pediatric flatfoot between China and Afghanistan. By demonstrating that standardized measurement is feasible even in resource-limited settings such as Kandahar, it highlights both the research value and the practical benefit of such work.

## Data Availability

The original contributions presented in the study are included in the article/Supplementary Material; further inquiries can be directed to the corresponding authors.

## References

[1] Farris DJ Kelly LA Cresswell AG Lichtwark GA. The functional importance of human foot muscles for bipedal locomotion. Proc Natl Acad Sci USA. (2019) 116:1645–50. 10.1073/pnas.1812820116 30655349 PMC6358692

[2] Liu Q Zhao C Yang X Tang J Chen J Tang L Biomechanics of transverse axis of medial longitudinal arch of children’s foot based on 3D scanning. Front Pediatr. (2023) 11:1197439. 10.3389/fped.2023.1197439 37492612 PMC10364607

[3] Birhanu A Nagarchi K Getahun F Gebremichael MA Wondmagegn H. Magnitude of flat foot and its associated factors among school-aged children in Southern Ethiopia: an institution-based cross-sectional study. BMC Musculoskelet Disord. (2023) 24:966. 10.1186/s12891-023-07082-6 38093248 PMC10716928

[4] Carr JB Yang S Lather LA. Pediatric pes planus: a state-of-the-art review. Pediatrics. (2016) 137:e20151230. 10.1542/peds.2015-1230 26908688

[5] Alkhreisat M Battah K Aldahamsheh O Alananzh M Ababneh O Farouji I. Novel presentation of rigid flat foot flexor hallucis longus passing through the subtalar joint: a comprehensive case report. Radiol Case Rep. (2024) 19:5187–90. 10.1016/j.radcr.2024.07.106 39263505 PMC11388039

[6] Kardm SM Alanazi ZA Aldugman TAS Reddy RS Gautam AP. Prevalence and functional impact of flexible flatfoot in school-aged children: a cross-sectional clinical and postural assessment. J Orthop Surg Res. (2025) 20:783. 10.1186/s13018-025-06207-y 40841947 PMC12369048

[7] Giuca G Marletta DA Zampogna B Sanzarello I Nanni M Leonetti D. Correlation between the severity of flatfoot and risk factors in children and adolescents: a systematic review. Osteology. (2025) 5:11. 10.3390/osteology5020011

[8] Aenumulapalli A. Prevalence of flexible flat foot in adults: a cross-sectional study. JCDR. (2017) 11:AC17. 10.7860/JCDR/2017/26566.10059 28764143 PMC5535336

[9] Ambatkar SY Ambade R. Foot print analysis and prevalence of flat foot among children of rural India—a study protocol. JPRI. (2021) 33:906–11. 10.9734/jpri/2021/v33i60B34693

[10] Monestier L Riva G Latiff M Marciandi L Bozzi E Pelozzi A Pediatric flexible flatfoot: does obesity influence the outcomes of arthroereisis? World J Orthop. (2024) 15:850–7. 10.5312/wjo.v15.i9.850 39318489 PMC11417632

[11] Xu L Gu H Zhang Y Sun T Yu J. Risk factors of flatfoot in children: a systematic review and meta-analysis. Int J Environ Res Public Health. (2022) 19:8247. 10.3390/ijerph19148247 35886097 PMC9319536

[12] Williams CM Banwell HA Paterson KL Gobbi K Burton S Hill M Parents, health professionals and footwear stakeholders’ beliefs on the importance of different features of young children’s footwear: a qualitative study. J Foot Ankle Res. (2022) 15:73. 10.1186/s13047-022-00580-1 36224579 PMC9559837

[13] Zhang Y Wu T Huang J Zhang Z Liu L Xiong Y Screening analysis of flat foot disease among school-age children in Kunming city. Chin J Sch Health. (2023) 44:765. 10.16835/j.cnki.1000-9817.2023.05.028

[14] Abich Y Mihiret T Yihunie Akalu T Gashaw M Janakiraman B. Flatfoot and associated factors among Ethiopian school children aged 11 to 15 years: a school-based study. PLoS One. (2020) 15:e0238001. 10.1371/journal.pone.0238001 32841276 PMC7447044

[15] Zhang N Ma G. Interpretation of WHO guideline: assessing and managing children at primary health-care facilities to prevent overweight and obesity in the context of the double burden of malnutrition. Glob Health J. (2018) 2:1–13. 10.1016/S2414-6447(19)30136-8 29578661

[16] Sadeghi-Demneh E Jafarian F Melvin JMA Azadinia F Shamsi F Jafarpishe M. Flatfoot in school-age children: prevalence and associated factors. Foot Ankle Spec. (2015) 8:186–93. 10.1177/1938640015578520 25819811

[17] Büyükçelebi H Açak M Eken Ö Doğaner A Özen G Ardigò LP. Association between pediatric obesity and foot morphology: insights from a large-scale cross-sectional study using photogrammetry. BMC Pediatr. (2025) 25:628. 10.1186/s12887-025-05966-1 40818938 PMC12357437

[18] Hollander K de Villiers JE Sehner S Wegscheider K Braumann K-M Venter R Growing-up (habitually) barefoot influences the development of foot and arch morphology in children and adolescents. Sci Rep. (2017) 7:8079. 10.1038/s41598-017-07868-4 28808276 PMC5556098

[19] Žukauskas S Barauskas V Degliūtė-Muller R Čekanauskas E. Really asymptomatic? Health-related quality of life and objective clinical foot characteristics among 5–10-year-old children with a flexible flatfoot. J Clin Med. (2023) 12:3331. 10.3390/jcm12093331 37176771 PMC10179374

[20] Oerlemans LNT Peeters CMM Munnik-Hagewoud R Nijholt IM Witlox A Verheyen CCPM. Foot orthoses for flexible flatfeet in children and adults: a systematic review and meta-analysis of patient-reported outcomes. BMC Musculoskelet Disord. (2023) 24:16. 10.1186/s12891-022-06044-8 36611153 PMC9825043

[21] Vergara-Amador E Serrano Sánchez RF Correa Posada JR Molano AC Guevara OA. Prevalence of flatfoot in school between 3 and 10 years. Study of two different populations geographically and socially. Colomb Med (Cali). (2012) 43:141–6. 10.25100/cm.v43i2.785 24893055 PMC4001940

[22] Alsuhaymi A Almohammadi F Alharbi O Alawfi A Olfat M Alhazmi O Flatfoot among school-age children in almadinah almunawwarah: prevalence and risk factors. J Musculoskelet Surg Res. (2019) 3:204. 10.4103/jmsr.jmsr_89_18

[23] Chang J-H Wang S-H Kuo C-L Shen HC Hong Y-W Lin L-C. Prevalence of flexible flatfoot in Taiwanese school-aged children in relation to obesity, gender, and age. Eur J Pediatr. (2010) 169:447–52. 10.1007/s00431-009-1050-9 19756732

[24] Fong Yan A Quinlan S Cheung RTH. Minimalist school shoes improve intrinsic foot muscle size, strength, and arch integrity among primary school students. J Sports Sci. (2024) 42:1157–63. 10.1080/02640414.2024.2386213 39087807

